# Biochemical Characterization of the Rice Cinnamyl Alcohol Dehydrogenase Gene Family

**DOI:** 10.3390/molecules23102659

**Published:** 2018-10-16

**Authors:** Hye Lin Park, Tae Lim Kim, Seong Hee Bhoo, Tae Hoon Lee, Sang-Won Lee, Man-Ho Cho

**Affiliations:** 1 Graduate School of Biotechnology and College of Life Science, Kyung Hee University, Yongin 17104, Korea; hlpark@khu.ac.kr (H.L.P.); ktlmi@khu.ac.kr (T.L.K.); shbhoo@khu.ac.kr (S.H.B.); 2 Department of Applied Chemistry, Kyung Hee University, Yongin 17104, Korea; thlee@khu.ac.kr

**Keywords:** cinnamyl alcohol dehydrogenase, rice, phenylpropanoid pathway, hydroxycinnamyl alcohol, lignin

## Abstract

Cinnamyl alcohol dehydrogenase (CAD) is involved in the final step of the phenylpropanod pathway, catalyzing the NADPH-dependent reduction of hydroxy-cinnamaldehydes into the corresponding alcohols. The rice genome contains twelve *CAD* and *CAD-like* genes, collectively called *OsCAD*s. To elucidate the biochemical function of the *OsCAD*s, *OsCAD1*, *2*, *6*, and *7*, which are highly expressed in rice, were cloned from rice tissues. The cloned *OsCAD*s were heterologously expressed in *Escherichia coli* as His-tag fusion proteins. The activity assay of the recombinant OsCADs showed that OsCAD2, 6, and 7 have CAD activity toward hydroxycinnamaldehydes, but OsCAD1 has no detectable catalytic activity. The kinetic parameters of the enzyme reactions demonstrated that *OsCAD2* has the highest catalytic activity among the examined enzymes. This result agrees well with the finding that the Zn binding and NADPH binding motifs and the residues constituting the substrate binding pocket in *bona fide* plant CADs were fully conserved in *OsCAD2*. Although they have large variations in the residue for the substrate binding pocket, OsCAD6 and 7 catalyzed the reduction of hydroxycinnamaldehydes with a similar efficiency. Alignment of amino acid sequences showed that OsCAD1 lacks the GxxxxP motif for NADPH binding and has mismatches in residues important in the reduction process, which could be responsible for the loss of catalytic activity. OsCAD2 belongs to CAD Class I with *bona fide* CADs from other plant species and is constitutively expressed throughout the developmental stages of rice, with preferential expression in actively lignifying tissues such as the root, stem, and panicle, suggesting that it is mainly involved in developmental lignification in rice. The expression of *OsCAD2* was also induced by biotic and abiotic stresses such as *Xanthomonas oryzae* pv. *oryzae* (*Xoo*) infection and UV-irradiation, suggesting that it plays a role in the defense response of rice, in addition to a *bona fide* role in developmental lignification. *OsCAD6* and 7 belong in CAD Class II. Their expression is relatively lower than that of *OsCAD2* and is confined to certain tissues, such as the leaf sheath, stem, and panicle. The expression of *OsCAD6* was stimulated by *Xoo* infection and UV-irradiation. Thus *OsCAD6* appears to be an inducible OsCAD that is likely involved in the defense response of rice against biotic and abiotic stresses.

## 1. Introduction

Hydroxycinnamyl alcohols are the final metabolites in the phenylpropanoid pathway and serve as precursors for lignin polymers and diverse phenolic compounds, such as lignans and phenylpropenes. Lignin is a complex phenolic polymer mainly derived from *p*-coumaryl alcohol, coniferyl alcohol, and sinapyl alcohol (called monolignols) [1,2]. Lignin is primarily deposited in the secondary cell walls of xylem and fiber cells in vascular plants, providing hydrophobicity and rigidity to the cell walls for water conduction and mechanical support [2,3,4,5]. Because of its complexity and physical toughness, lignin also plays an important role in plant defense, and thus its synthesis and deposition can be induced by biotic and abiotic stresses [3,6,7,8]. Coniferyl alcohol and its glycosylated derivatives have been found to accumulate and be involved in the defense against fungal pathogens [9]. Lignans are defense-related phenolic compounds, typically dimers of hydroxycinnamyl alcohols formed by the oxidative coupling of the monomers [1,10,11,12]. In *Arabidopsis*, the lignans lariciresinol and pinoresinol were found to be accumulated in response to an infection of the fungal pathogen *Verticillium longisporum*, suggesting their defensive role [9]. Lignans also have diverse health-beneficial properties, including anti-oxidative, anti-viral, anti-inflammatory, and anti-tumor activities [1,12,13,14]. Podophyllotoxin is a therapeutic lignan originally isolated in *Podophyllum* and used to treat many types of tumors, including genital warts [15,16]. In addition to serving as the monomers for lignin and lignans, hydroxycinnamoyl alcohols are metabolic intermediates for diverse phenylpropanoids, including phenylpropenes [17,18,19,20]. Phenylpropenes such as eugenol, isoeugenol and anethole are volatile ingredients in plant essential oils and involved in plant defenses, with anti-microbial and insect-repelling effects [19,20,21,22].

Hydroxycinnamyl alcohols are biosynthesized from Phe through the phenylpropanoid pathway [8,23,24]. CAD participates in the last step of the phenylpropanoid pathway by catalyzing the NADPH-dependent reduction of hydroxycinnamaldehydes into the corresponding alcohols [8,23,24]. In plants, *CAD* genes exist as a small gene family, with nine members in *Arabidopsis thaliana*, 14 genes in sorghum (*Sorghum bicolor*), 15 genes in *Populus trichocarpa*, and 12 genes in wheat (*Triticum aestivum*) [25,26,27,28,29]. Biochemical characterizations, expression analyses and mutant studies have shown that one or two *CAD* genes are mainly responsible for developmental lignification, with the others having different functions in plants [25,26,27,28,30,31,32]. In *Arabidopsis*, two *AtCAD*s were found to encode highly active proteins, and their simultaneous mutation resulted in the drastic reduction of lignin content in the floral stem [26,33].

In rice, twelve genes have been identified as *CAD*s and named *OsCAD1-7*, *8A-D*, and *9* [34,35]. *OsCAD*s are distributed across the rice chromosomes. *OsCAD1* and *3* are existed in chromosome 10, and *OsCAD6* and 7 are located on chromosome 4. Chromosomes 2, 3, 8 and 11 include *OsCAD2*, *9*, *5* and *4*, respectively. The closely-related *OsCAD8A*-*D* genes are localized at the same locus on chromosome 9 [34,35]. These genes suggested to be created by a recent duplication followed by inversion, and other *OsCAD*s are present in single copies in rice chromosomes [34]. Promoter::β-glucuronidase (GUS) and RNA blot analysis showed that OsCAD2 was strongly expressed in lignifying tissues in rice [34,35]. So far, only a few studies have suggested the function of the *OsCAD*s. The lignin-deficient phenotype of the *gold hull and internode2* (*gh2*) mutant and the expression of the *GH2* gene in lignifying tissues suggests that *OsCAD2* is the major *CAD* gene in rice [35,36]. Analysis of the *flexible culm*1 (*fc1*) mutant suggests the complementary role of OsCAD7 in lignification [37]. To elucidate biochemical function of *OsCAD*s, we cloned *OsCAD1*, *2*, *6*, and *7*, which are all expressed substantially in rice plants. Recombinant OsCAD1, 2, 6 and 7 proteins were produced in *E. coli*, and their catalytic properties toward hydroxycinnamaldehyde substrates were examined. Of the examined OsCADs, OsCAD 2, 6, and 7 were found to have CAD activity, with OsCAD2 being the most active. To delineate the physiological role of the *OsCAD*s, we analyzed their expression in different tissues and developmental stages. Stress-responsive changes in *OsCAD* expression were also examined.

## 2. Results

### 2.1. The CAD Gene Family in Rice

Gene information about the twelve *OsCAD*s was retrieved from the MSU Rice Genome Annotation Project (RGAP) database [38]. The lengths of the open reading frame (ORF) of most *OsCAD*s (except *OsCAD5* and *OsCAD8B* and *C*) are 1032–1140 nucleotides, encoding polypeptides 343–379 amino acids long (Table 1), which is similar to the *bona fide* CADs from *Arabidopsis*, maize, wheat, sorghum, and *Populus*, which range from 1068 to 1131 nucleotides [25,26,27,28,30,31]. *OsCAD5* has an ORF of 999 nucleotides encoding a polypeptide of 332 amino acids (Table 1 and Figure S1). A previous study reported that the *OsCAD5* gene encodes a shorter polypeptide than the other OsCADs because of a stop codon at amino acid 297 [34]. The ORF lengths of *OsCAD8B* and *C* are slightly longer than those of the typical *CAD*s because they contain N-terminal extensions (Supplementary Figure S1). The gene information retrieved from the Rice Annotation Project Database (RAP-DB) [39] showed no N-terminal extensions in *OsCAD8B* and *C* [35]. This discrepancy is caused by to the different predictions of start codons for *OsCAD8B* and *C* in the different databases.

**Figure 1:**
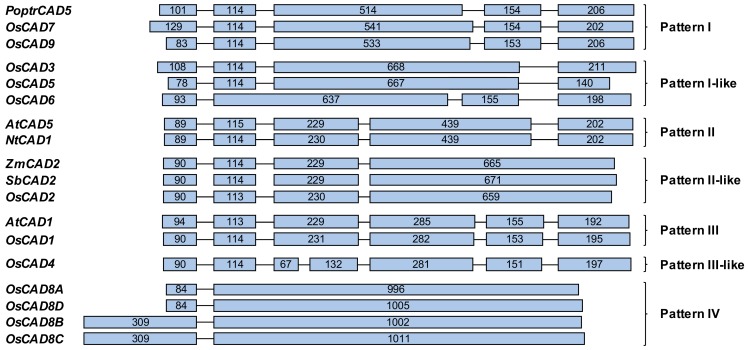
The exon-intron structures of the *OsCADs* and other plant *CAD* genes. Exons and introns are indicated by boxes and lines, respectively. The numbers in the boxes indicate the exon sizes, and the intron sizes are not to scale. The exon-intron patterns of the plant CADs are indicated on the right side. *A. thaliana CAD* (*AtCAD*), *Z. mays CAD* (*ZmCAD*), *P. trichocarpa CAD* (*PoptrCAD*), *S. bicolor CAD* (*SbCAD*) and *N. tabacum CAD* (*NtCAD*).

*OsCAD*s are composed of between two and seven exons (Figure 1). Barakat et al. [28] divided the exon-intron structures of the *PoptrCAD*s from *P. trichocarpa* into three patterns, Patterns I to III. In addition to those patterns, four more exon-intron patterns were found in the *OsCAD*s (Figure 1). The exon-intron structures of the known *bona fide* CADs belong in Pattern II or Pattern II-like. Pattern II is composed of five exons and contains dicot *bona fide* CADs, including *AtCAD5* and *NtCAD1* from tobacco (*Nicotiana tabacum*). The exon-intron structures of the monocot *bona fide* CADs, such as *ZmCAD2* and *SbCAD2*, are similar to those of Pattern II and designated as Pattern II-like (Figure 1). In Pattern II-like, the fourth and fifth exons of Pattern II are combined into one exon. *OsCAD2* shows the Pattern II-like exon-intron structure (Figure 1). *OsCAD7* and *9* show the exon-intron structure of Pattern I, which contains *CAD*s from monocots and eudicots (Figure 1) [28]. The exon-intron structures of *OsCAD3, 5*, and *6* are similar to Pattern I and designated as Pattern I-like (Figure 1). Some *CAD*s from monocots, eudicots and gymnosperms are composed of six exons and classified as Pattern III [28]. It has been reported that *CAD*s belonging to Pattern III, such as *AtCAD1* and *PtoCAD12*, encode proteins having almost no CAD activity [26,32]. *OsCAD1* is classified as Pattern III. Although *OsCAD4* has seven exons, it shows structures similar to Pattern III, so it is classified as Pattern III-like (Figure 1). The closely related *OsCAD8A*–*D* have two exons, and their second exons are exceptionally long, so they ate classified as Pattern IV (Figure 1).

### 2.2. Sequence Homology and Phylogenetic Analysis of the OsCADs

**Figure 2:**
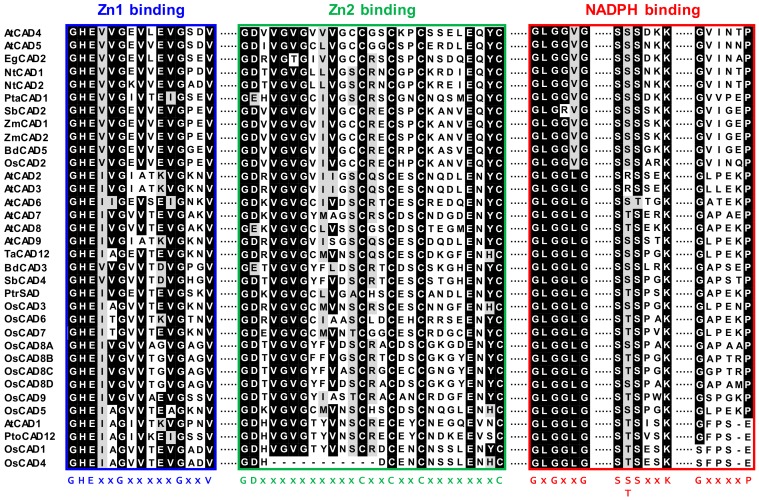
The conserved Zn binding motifs and NADPH binding consensus in plant CADs. Multiple alignment of the amino acid sequences in the OsCADs and other plant CADs was performed and the conserved motifs are presented. The consensus sequences are indicated below the alignments (x indicates any amino acid). AtCAD1-9 from *A. thaliana*, EgCAD2 from *E. gunnii*, NtCAD1 and 2 from *N. tabacum*, PtaCAD1 from *P. taeda*, SbCAD2 and 4 from *S. bicolor*, ZmCAD1 and 2 from *Z. mays,* BdCAD3 and 5 from *B. distachyon*, TaCAD12 from *T. aestivum*, PtoCAD12 from *P. tomentosa*, and PtrSAD from *P. tremuloides*.

Multiple alignments of CAD protein sequences revealed that OsCAD1-9 had 54–93% similarity to *bona fide* CADs from other plant species (Supplementary Table S1). OsCAD2 was highly homologous, with 81–93% similarity, and the others showed 54–68% similarity to the *bona fide* CADs (Supplementary Table S1). As members of the medium chain dehydrogenase/reductase (MDR) superfamily, plant CADs contain highly conserved Zn binding motifs (Zn1 binding motif for catalytic Zn^2+^ and Zn2 binding motif for structural Zn^2+^) and NADPH binding signatures [34,35,40]. These signature motifs are well conserved in most OsCADs (Figure 2 and Supplementary Figure S1). The Zn1 binding consensus sequence of GHE(x)_2_G(x)_5_G(x)_2_V is conserved in all OsCADs. In addition to the Zn1 consensus residues, the C47, H69, E70, and C163 residues coordinated with the catalytic Zn^2+^ ion in AtCAD5 are conserved in all OsCADs (Supplementary Figure S1). The GD(x)_10_C(x)_2_C(x)_2_C(x)_7_C consensus of the Zn2 binding motif is conserved in most OsCADs except OsCAD4. Amino acid alignment showed that eleven residues in the Zn2 binding motif are deleted in OsCAD4 (Figure 2 and Supplementary Figure S1). This deletion led to missing the Cys residue corresponding to the C100 of AtCAD5, which is coordinated with the structural Zn^2+^ ion together with the C103, C106, and C114 residues [40]. Three flexible loops, each containing the GxxGxxG, S(S/T)SxxK, and GxxxxP consensus, were suggested to be involved in NADPH binding by CADs [40]. These motifs are conserved in most OsCADs, although OsCAD1 and 4 have no GxxxxP consensus (Figure 2 and Supplementary Figure S1).

Many studies have shown that CADs are dimeric enzymes [40,41,42]. The crystal structure of AtCAD5 demonstrates that the substrate binding pocket is composed of twelve residues (nine residues from one subunit and three residues from another subunit) and located in the cleft between two subunits [40]. In most OsCADs, these residues are conserved or changed for other amino acids with similar properties, but OsCAD1, 4, and 5 have significant variations in the substrate binding pocket (Supplementary Figure S1). The well-conserved T49 residue in AtCAD5 is changed to Ala in OsCAD1 and 4. Variations of the M60 in AtCAD5 with uncharged amino acids were frequently found in other catalytically active CADs, whereas this residue was changed to Asp in OsCAD4. OsCAD4 also lacks the amino acid found at the C95 position of AtCAD5. Both amino acid sequences of OsCAD5 (obtained from the MSU RGAP database and the RAP-DB) showed large deletions in the C-terminal region, leading to the absence of three residues corresponding to the L290, F299, and I300 of AtCAD5 (Supplementary Figure S1) [35]. In addition to the conserved motifs and residues constituting the substrate binding pocket, the H52 residue in AtCAD5 is well conserved among the CADs, which is important in the CAD activity of participating in the proton shuttle system during catalysis [40]. This His residue was found to be changed to Ala and Ile in OsCAD1 and 4, respectively (Supplementary Figure S1).

Previous phylogenetic analyses have demonstrated that plant CADs are divided into three classes, Class I, II, and III [28,34,35,43,44]. Class I comprises of the *bona fide* CADs, including OsCAD2 (Supplementary Figure S2). Although the plant CAD families are composed of about ten genes, only one or two members from each have been grouped into Class I except in cassava (*Manihot esculenta*) [43]. Accumulating evidence shows that CAD members in Class I participate in lignin deposition in the secondary cell walls during plant growth and development [25,26,27,28,30,40,42,45]. A large number of CAD members belong to Class II, which contains all OsCADs except OsCAD2, 1, and 4 (Supplementary Figure S2) [32,43]. CADs included in this group, such as TaCAD12 from wheat; PtoCAD2, 3, and 8 from *P. tomentosa*; AtCAD2, 3, 7 and 8 from *Arabidopsis*; and SbCAD4 from sorghum, have been shown to encode catalytically active CAD proteins [26,29,32,46]. Class III is a small group of CADs, including OsCAD1 and 4, AtCAD1, and PtoCAD12 (Supplementary Figure S2) [26,32,43].

### 2.3. Heterologous Expression and Biochemical Characterization of OsCADs

To elucidate the biochemical functions of OsCADs, we cloned *OsCAD* cDNA from wild-type rice plants. The cDNA of *OsCAD1*, *2*, *6*, and *7* were cloned from rice leaves. Despite extensive trials to clone other *OsCAD*s from plant samples of different tissues from developmental stages of rice, we could not clone *OsCAD3*, *4*, *5*, *8A*-*D* or *9*, probably because of their very low expression throughout all developmental stages (Supplementary Figure S3). It was previously reported that *OsCAD2* was most abundantly expressed in rice culm. *OsCAD1* was also substantially expressed in rice culm. However, transcripts of the other *OsCAD*s were not detected or were only at very low levels in rice culm [35].

**Figure 3:**
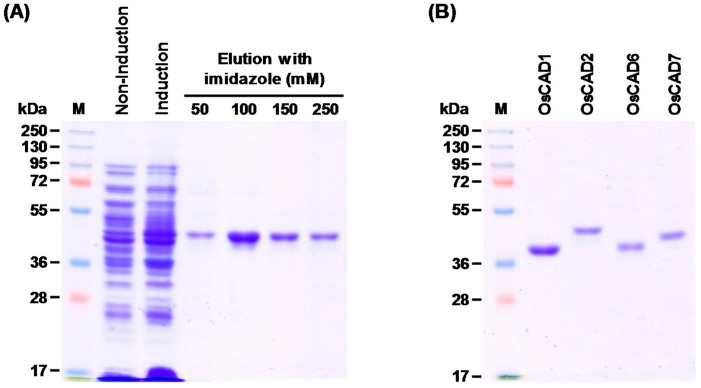
Purification of recombinant OsCADs expressed in *E. coli*. The His-tagged OsCAD1, 2, 6 and 7 were expressed in *E. coli* in a soluble form. The recombinant proteins were purified by Ni^2+^-affinity chromatography. (**A**) Representative purification of the recombinant OsCAD2 protein. (**B**) Affinity purified recombinant OsCAD proteins. M, Molecular weight marker.

We tried to heterologously express the cloned *OsCAD*s in *E. coli* in a His-tagged form. OsCAD1, 2, 6, and 7 were successfully expressed as soluble protein in *E. coli*. The His-tagged OsCAD proteins were purified with Ni^2+^ affinity chromatography to apparent homogeneity (Figure 3). The molecular masses of the recombinant OsCAD proteins, determined on SDS-PAGE, were 41.0 to 46.5 kDa, which agrees well with their theoretical molecular masses (Table 1 and Figure 3B).

**Figure 4:**
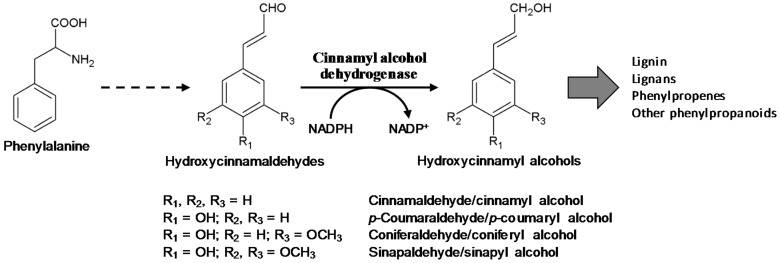
Cinnamyl alcohol dehydrogenase catalyzed reactions. The hydroxycinnamaldehyde substrates used in this study and the corresponding hydroxycinnamyl alcohols are presented.

The enzyme activity of the recombinant OsCADs was assayed using four hydroxycinnamaldehyde substrates; *p*-coumaraldehyde, coniferaldehyde, sinapaldehyde and cinnamaldehyde (Figure 4). OsCAD2, 6, and 7 showed NADPH-dependent reductase activity toward the examined substrates (Table 2). In these OsCADs, the signature motifs and amino acid residues involved in substrate binding and catalytic activity are well conserved (Figure 2 and Supplementary Figure S1). However, OsCAD1 had no detectable activity toward the hydroxycinnamaldehyde substrates. As mentioned above, OsCAD1 has mismatches in the conserved amino acid residues, including S/T49A and H52A, and a lack of the GxxxxP motif for NADPH binding, which may lead to the loss of catalytic activity (Figure 2 and Supplementary Figure S1). Similar variations were also found in OsCAD4, PtoCAD12, and AtCAD1 (Figure 2 and Supplementary Figure S1). In addition, the deletion of eleven amino acids from the Zn2 binding motif was found in OsCAD4.

The kinetic parameters of the recombinant OsCAD2, 6 and 7 catalyzed reactions toward the hydroxycinnamaldehyde substrates were determined (Table 2). The *K*_M_ values of OsCAD2 for *p*-coumaraldehyde, coniferaldehyde, sinapaldehyde, and cinnamaldehyde were between 4.37 and 9.36 μM, indicating that it has a similar affinity to the examined substrates. The *k*_cat_/*K*_M_ values of OsCAD2 for coniferaldehyde and sinapaldehyde were three to four-fold higher than for *p*-coumaraldehyde and cinnamaldehyde, indicating that it has a greater catalytic efficiency toward coniferaldehyde and sinapaldehyde than toward the other substrates (Table 2). The catalytic efficiency of OsCAD2 toward coniferaldehyde was 1.34-fold higher than that toward sinapaldehyde, which is consistent with the previously kinetic property of the recombinant OsCAD2 with 1.78-fold higher catalytic efficiency toward coniferaldehyde than toward sinapaldehyde [36]. OsCAD6 and 7 have similar *K*_M_ values toward the examined substrates, showing less than an order of difference (10.72–28.25 μM for OsCAD6 and 2.66–14.11 μM for OsCAD7) (Table 2). The *K*_M_ values of OsCAD6 and 7 were also comparable with those of OsCAD2. By contrast, the *V*_max_ values of OsCAD6 and 7 were much lower than those of OsCAD2, with values more than a hundred-times less (Table 2). Therefore, the catalytic efficiencies (*k*_cat_/*K*_M_ values) of OsCAD2 are more than a hundred-times higher than those of OsCAD6 and 7. These results indicates that OsCAD6 and 7 are less catalytically active than OsCAD2, even though they have a similar affinity toward the hydroxycinnamaldehyde substrates.

### 2.4. Expression Analysis of the Biochemically Active OsCADs

**Figure 5:**
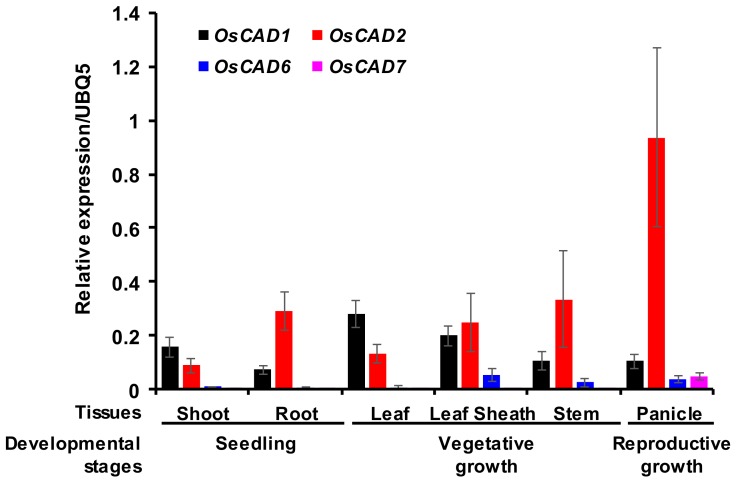
Expression of *OsCAD1*, *2*, *6*, and 7 in rice tissues from different developmental stages. The expression levels of the *OsCAD*s were examined by qRT-PCR analysis using specific primers (Supplementary Table S2). A ubiquitin gene (*OsUBQ5*) was amplified and used as an internal control. The expression levels of each *OsCAD* gene are presented as the relative expression compared to the *OsUBQ5* transcript levels. qRT-PCR analysis was performed on triplicate biological samples.

To delineate the physiological role of the *OsCAD*s encoding biochemically active proteins, we examined their expression levels by quantitative real-time PCR (qRT-PCR) analysis using total RNA extracted from rice tissues at different developmental stages. *OsCAD2* was constitutively expressed through all developmental stages (Figure 5). In particular, expression of *OsCAD2* was much higher in actively lignifying tissues such as the root, stem, and panicle. This result agrees well with the inclusion of OsCAD2 in Class I in the phylogeny, along with other *bona fide* CAD*s* (Supplementary Figure S2). Hirano et al. [35] also reported that among *OsCAD*s, *OsCAD2* is most abundantly expressed in the uppermost internode of cv. *Nipponbare* at the heading stage. In addition, *OsCAD2* was reported to be highly expressed in the root tissue [34]. In the *OsCAD2* promoter::GUS fusion analysis, GUS activity was detected in the vascular bundles and lateral veins in rice leaf sheath and blades [34]. The other *OsCAD*s showed very low expression levels in the root, stem, and panicle tissues compared with *OsCAD2* (Supplementary Figure S3). Although the *OsCAD6* transcript was detected in all the tissues examined, the expression levels in the shoot, root, and leaf were near the detection limit of the analysis, and the abundance in the leaf sheath, stem, and panicle tissues was much less than that of *OsCAD2* (Figure 5). The *OsCAD7* transcript was detected only in the panicle tissue with less abundance than *OsCAD2* (Figure 5). This result suggests that *OsCAD6* and *7* play a limited function in certain developmental stages and physiological conditions. Although the recombinant proteins showed no detectable activity, *OsCAD1* was substantially expressed in all the examined tissues (Figure 5 and Supplementary Figure S3). This result agrees well with the expression of *OsCAD1* and *2* in rice culm, which showed the highest expression of *OsCAD2* and no or very low expression of the other *OsCAD*s [35].

**Figure 6:**
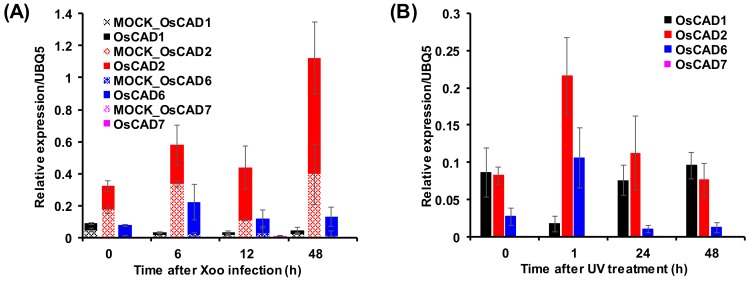
Expression of *OsCAD1*, *2*, *6*, and 7 in response to *Xoo* infection (**A**) and UV-irradiation (**B**). The expression levels of the *OsCAD*s were examined by qRT-PCR analysis using specific primers (Supplementary Table S2). A ubiquitin gene (*OsUBQ5*) was amplified and used as an internal control. The expression levels of each *OsCAD* gene are presented as the relative expression compared to the *OsUBQ5* transcript levels. qRT-PCR analysis was performed on triplicate biological samples.

It is well known that the expression of phenylpropanoid pathway genes is up-regulated in response to biotic and abiotic stresses [47,48]. An in silico transcriptomic analysis of *OsCAD*s using microarray data from the Genevestigator database showed that the expression of *OsCAD2* and *6*, encoding biochemically active CADs, increased more than two-folds in rice leaves infected with *M. grisea* and *X. orizae* pv. *oryzicola* (*Xoc*) (Figure S4). *Xoo* infection stimulated the expression of *OsCAD2, 8A*, and *8C* (Figure S4). qRT-PCR analysis of *OsCAD*s in *Xoo* infected rice leaves was performed to ascertain biotic stress-induced expression of the biochemically functional *OsCAD*s. The qRT-PCR analysis showed that the expression of *OsCAD2* and *6* were stimulated by *Xoo* infection (Figure 6A). OsCAD6 and 2 showed the highest expression levels 6 and 48 h after *Xoo* infection, respectively (Figure 6A). The expression of *OsCAD7* and *1* was not significantly changed by *Xoo* infection (Figure 6A). A previous study reported that *M. grisea*, *Xoo*, and *Xoc* infections induced rice cinnamoyl-CoA reductases (*OsCCR*s) involved in the formation of hydroxycinnamaldehydes [48]. In addition to pathogen infections, the expression of functional *OsCCRs* was also stimulated by abiotic stresses such as UV irradiation [48]. The microarray data from UV-treated rice leaves obtained in our previous study [49] showed that *OsCAD2*, *6*, and *8A* were immediately up-regulated by UV-irradiation (Figure S5A). The qRT-PCR analysis of the biochemically functional *OsCAD*s confirmed the UV-stimulated expression of *OsCAD2* and *6* (Figure 6B). An in silico transcriptomic analysis of public microarray data indicated that expression of *OsCAD3* and *8A* was also induced by wounding or cold treatment (Figure S5B). These findings suggest that *OsCAD2*, 3, *6*, and *8A* likely participate in defense responses to diverse biotic and abiotic stresses.

## 3. Discussion

Catalyzing the last step of the phenylpropanoid pathway, CAD is primarily involved in the deposition of lignin in the secondary cell walls and also participates in plant defenses by mediating the stress-induced lignification and synthesis of phenolic compounds [3,6,7,8]. The functions of CAD family members have been studied in many plant species, including *Arabidopsis* [25,26], wheat [31], maize [30,45], *P. tomentosa* [32], *P. trichocarpa* [28], and sorghum [2], and it is generally found that one or two CADs play a *bona fide* role in developmental lignification. In the present study, we cloned *OsCAD1, 2, 6*, and *7*, which are substantially expressed in rice tissues, and then heterologously expressed them in *E. coli* to elucidate the biochemical functions of the OsCADs. The enzyme activity assay of the recombinant OsCADs showed that OsCAD2, 6, and 7 have CAD activity, with OsCAD2 showing a much greater catalytic efficiency toward the examined substrates, particularly coniferaldehyde and sinapaldehyde, than OsCAD6 and 7 (Table 2). The catalytic efficiency of OsCAD2, which had *k*_cat_/*K*_M_ values of 92.43–429.29 μM^−1^ min^−1^ toward the hydroxycinnamaldehyde substrates, was comparable to those of the other *bona fide* plant CADs. The *k*_cat_/*K*_M_ values of *bona fide* CADs, such as AtCAD5, PtoCAD1, BdCAD5, SbCAD2 (Bmr6), and TaCAD1, toward hydroxycinnamaldehydes were ranging from 15.01 to 772.2 μM^−1^ min^−1^ [26,31,32,46,50]. The crystal structure of the AtCAD5 protein demonstrated that twelve residues, mostly hydrophobic amino acids, constitute the substrate binding pocket [40]. It was suggested that nine residues (Q53, L58, M60, W119, V216, P286, L290, F299, and I300) are completely conserved among all the *bona fide* CADs, and two residues have conservative heterogeneity (Thr or Ser for residue 49, and Met or Ile for residue 289). Residue 95 was found to be variable in *bona fide* CADs [40]. However, the M60 residue of AtCAD5 was found to be replaced by Ala in monocot CADs such as SbCAD2, BdCAD5, and ZmCAD1 and 2, which is likely a property of the *bona fide* monocot CADs (Supplementary Figure S1). The amino acid residues constituting the substrate binding pocket are entirely conserved in OsCAD2 with the monocot-characteristic M60A variation (Supplementary Figure S1). This finding is consistent with OsCAD2 having the highest catalytic activity among the examined OsCADs (Table 2).

The qRT-PCR analysis also showed that *OsCAD2* is constitutively expressed throughout all developmental stages, being most highly expressed in actively lignifying tissues (Figure 5). These findings, together with its high catalytic activity, grouping into exon-intron Pattern II-like, and phylogenetic Class I, all strongly support that OsCAD2 is the *bona fide* CAD in rice. This finding is also consistent with the lignin-deficient phenotype of the rice *gh2* mutant, which is caused by a point mutation in *OsCAD2* [35,36].

The residues constituting the substrate binding pocket in *bona fide* CADs, except for the T/S49 residue, are mostly replaced to uncharged and/or hydrophobic amino acids in the Class II CADs (Supplementary Figure S1). Of the Class II OsCADs, OsCAD3, 8A, 8D, and 9 showed a few variations with polar or charged amino acids in those residues (Figure S1). All the Class II CADs examined in the present study replaced the Leu in residue 58 with Trp, which might be a characteristic of the Class II CADs. By contrast, this residue is variable in the Class III CADs (Supplementary Figure S1). Despite the large variations, the enzyme activity assays showed that many Class II CADs, such as the AtCADs (AtCAD2, 3, 7, and 8), PtoCADs (PtoCAD2, 3, and 8), TaCAD12, BdCAD3, and SbCAD4, are catalytically active, even though they are much less active than their *bona fide* counterparts [26,29,32,46,50]. In OsCAD6 and 7, most residues for the substrate binding pocket are displaced by uncharged amino acids, including the L58W variation (Supplementary Figure S1). The activity assay of the recombinant proteins indeed showed that OsCAD 6 and 7 have CAD activity (Table 2), suggesting that Class II CADs with variations in the residues of the substrate binding pocket with uncharged amino acids possibly retain their catalytic activity.

OsCAD1 was found to lack the GxxxxP motif needed for NADPH binding and to have many mismatches in the residues of the substrate binding pocket, including T/S49A. The hydroxyl group in the T/S49 residue, together with the H52, was proposed to participate in the proton relay during the reduction process in CADs [40]. In OsCAD1, the H52 residue was changed to Ala. In AtCAD1 and PtoCAD12, the H52 was changed to Ile (Supplementary Figure S1). The Q53W, P286A, M/I289N, and I300T variations in OsCAD1 were also found in AtCAD1 and PtoCAD12 (Supplementary Figure S1). A biochemical study showed that AtCAD1 has almost no detectable CAD activity and PtoCAD12 has no catalytic activity [26,32], which agrees with our finding that OsCAD1 has no detectable CAD activity. Despite its no CAD activity, *OsCAD1* was highly expressed in all examined tissues over developmental stages (Figure 5). In *Arabidopsis*, *AtCAD1* expression was induced by the treatment of the allelochemical benzoxazolin-2(3*H*)-one and *AtCAD1* was suggested to be involved in detoxification processes of the allelochemical [51]. In was reported that *AaCAD* from *Artemisia annua* uses the monoterpene citral as a substrate in addition to hydroxycinnamaldehyde substrates [52]. *MsaCAD1* from alfalfa (*Medicago sativa*) showed reductase activity toward valeraldehyde and benzoaldehydes [53]. As a member of the MDR superfamily, the bacterial CAD from *Helicobacter pylori* used a broad range of substrates, which include hydroxycinnamaldehydes, benzaldehyde, and acetaldehyde [54]. To elucidate the physiological role of *OsCAD1*, further studies including the delineation of substrate spectrum are needed to be performed.

In addition to lack of the GxxxxP motif and the T/S49A variation, OsCAD4 has the deletion of eleven residues in the Zn1 binding motif (Figure 2 and Supplementary Figure S1). Residue 60 of the substrate binding pocket was also found to be changed to the charged residue Asp in OsCAD4, AtCAD1, and PtoCAD12 (Supplementary Figure S1). These findings imply that OsCAD4 likely has no catalytic activity. OsCAD1 and 4, together with AtCAD1 and PtoCAD12, are in CAD Class III (Supplementary Figure S2). Although the Zn binding and NADPH binding motifs are well conserved, OsCAD5 has a large deletion in the C-terminal region, which results in the absence of the corresponding residues for L290, F299, and I300, which constitute the substrate binding pocket in AtCAD5 (Supplementary Figure S1) [40]. It was previously suggested that *OsCAD5* encoded a truncated, biochemically inactive protein [34,35].

Participating in two reductive steps from hydroxycinnamoyl-CoAs to monolignols, the substrate preferences of CCR and CAD have been thought to relate to the lignin composition of plants [3,31,48,55,56,57,58,59]. Lignin is mainly composed of guaiacyl (G)-, syringyl (S)-, and *p*-hydroxyphenyl (H)- units derived from coniferyl alcohol, sinapyl alcohol and *p*-coumaryl alcohol, respectively [2,3]. The lignins of gymnosperm wood are predominantly composed of G-units, but most angiosperm lignins are a mixture of G- and S-units, with a minor portion of H-units [2,3,4]. The *bona fide* CCRs have a substrate preference for feruloyl-CoA, a precursor for the G-unit, which is consistent with the lignin composition of plant species with higher G-content [48,60,61,62]. Enzyme activity assays have shown that the relationship between the substrate preference of the *bona fide* CADs and lignin composition was less strict than that for CCRs. PtCAD from aspen (*P. tremuloides*) and TaCAD1 from wheat showed more than a three-fold higher catalytic efficiency toward coniferaldehyde, a precursor for the G-unit, than toward *p*-coumaraldehyde and sinapaldehyde, which is consistent with the lignin compositions of those plants, which have a high content of G-units [31,59]. By contrast, AtCAD4 and 5 have the highest catalytic efficiency for *p*-coumaraldehyde, a precursor for the H-unit [26]. PtoCAD1 from *P. tomentosa* showed a similar catalytic activity for coniferaldehyde and sinapaldehyde [32]. In rice, the *bona fide* OsCCR20 showed the highest catalytic activity for feruloyl-CoA [48], whereas OsCAD2 was found to have a similar substrate preference for coniferaldehyde and sinapaldehyde (Table 2).

Although they are less efficient than OsCAD2, OsCAD6 and 7 showed catalytic activity toward the hydroxycinnamaldehydes without apparent substrate preferences (Table 2). It has commonly been found that the catalytic efficiency of CAD isozymes is much lower than that of the *bona fide* CADs [26,29,32,46,50]. Several studies have shown the complementary role of non-*bona fide* CADs in lignification. Although it had very low catalytic activity toward hydroxycinnamaldehydes, the increased expression of *AtCAD9* (named AtCAD1 by Eudes et al. [63]) partly restored the reduced lignin phenotype of the double mutant of *bona fide AtCAD*s [26,63]. In rice, the *fc1* mutant that is defective in *OsCAD7* showed reduction in culm strength, a decrease in CAD activity in the internodes and panicles, and reduced lignin content, suggesting the complementary role of *OsCAD7* in lignification [37].

In addition to their role in lignification, plant CADs participate in synthesizing of stress-related phenolic compounds [9,64]. In this regard, CAD expression has been found to be up-regulated by biotic and abiotic stresses. The induction of CAD gene expression by treatment with fungal elicitors was accompanied by an accumulation of the lignan lariciresinol in cell cultures of *Linum album* [64]. In *Arabidopsis*, infection with the fungal pathogen *V. longisporum* induced the expression of *AtCAD5* and *8*, together with *AtPAL1*, which led to an accumulation of hydroxycinnamyl alcohol derivatives such as coniferin, syringing, and lignans (lariciresinol glucosides and pinoresinol glucosides). These soluble phenylpropanoids could be involved in the defense response of *Arabidopsis* against *V. longisporum* infection [9]. The expression level of *TaCAD12* was found to be significantly higher in sharp eyespot-resistant wheat lines than in susceptible lines. Overexpression of *TaCAD12* resulted in enhanced resistance of transgenic wheat lines to sharp eye spot disease [29]. In rice, phenylpropanoid pathway genes have been induced in response to biotic and abiotic stresses [47,49,65,66]. The in silico transcriptomic analysis showed that *OsCAD2* expression was induced not only by *M. grisea*, *Xoo* and *Xoc* infections but also by UV and cold treatments (Figure S4 and Supplementary Figure S5). The qRT-PCR also showed that *Xoo* infection stimulated the expression of OsCAD2 (Figure 6A). This result suggests that, in addition to its role in lignification, *OsCAD2* is involved in defense response. We also found that the expression of *OsCAD6* and *8A* was stimulated by UV and cold treatments and pathogen infections (Figure 6 and Supplementary Figure S5), although their expression levels were very low during normal growth (Supplementary Figure S3) [34,35]. We therefore suggest that *OsCAD6* and *8A* are inducible and probably participate in defense mechanisms against biotic and abiotic stresses. Tobias and Chow [34] previously reported that among 12 *OsCADs*, only *OsCAD6* has peroxisomal targeting signal at the carboxy terminus. They suggested that *OsCAD6* was not involved directly in lignin biosynthesis, but might be participated in the biosynthesis of lignans [34].

Biotic and abiotic stresses are the primary factors in crop loss, which can cause more than 50% reduction of major crop yield [67,68,69,70]. Involving the lignin deposition in the secondary cell walls and biosynthesis of defense-related compounds, the phenylpropanoid pathway genes including *CAD*s are closely related with the resistance of crop plants against biotic and abiotic stresses [1,2,3,6,7,8,10,11,12]. As mentioned above, the overexpression of CAD also led to enhanced resistance of wheat plants to sharp eyespot [29]. Therefore, the understanding of biochemical and physiological functions of *OsCAD*s can provide a useful information for crop improvement.

## 4. Materials and Methods

### 4.1. Plant Growth and Materials

Wild-type rice (*Oryza sativa* L. spp. *Japonica* cv. *Dongjin*) seeds were sterilized with 50% bleach solution for 30 min, and washed with sterilized water more than five times. The sterilized seeds were germinated on Murashige and Skoog (MS) medium (Duchefa, Harlem, The Netherlands) containing 2% sucrose in a growth chamber with a 12 h day/12 h night photoperiod at 28 °C for one week. The one-week old seedlings were transferred to soil and grown in a greenhouse at 28 °C during the day and 20 °C at night. Root and shoot samples were collected from the rice seedlings grown on MS medium. Stem, leaf sheath and leaf samples were obtained from ten-week-old rice plants. Panicle samples were collected from 14-week-old rice plants. PXO99A (Philippine *Xoo* strain 6) compatible with *Dongjin* was used in this study. PXO99A strain was cultured at 28 °C on peptone sucrose agar plates for three days. The cultured PXO99A cells were resuspended with sterilized water for infection. Infection of *Xoo* strain PXO99A into rice leaves was carried out using the clipping method [71]. Mock treatment was also performed with sterilized water. Leaf samples were collected after 0, 6, 12, and 48 h after *Xoo* infection. UV-C treatment of rice plants was performed in a growth chamber with five 20 W germicidal lamps (maximum emission at 254 nm, 7.5 W UV output, Sankyo Denki Co., Kanagawa, Japan) for 2 h [49]. The UV-treated rice plants were transferred to a greenhouse, and the UV-treated rice leaves were collected 1, 24, 48, and 96 h after UV treatment. *p*-Coumaraldehyde, coniferaldehyde, sinapaldehyde, cinnamaldehyde and reduced *β*-nicotinamide adenine dinucleotide phosphate (NADPH) were purchased from Sigma-Aldrich (St. Louis, MO, USA). Reagents for buffers and other solutions were obtained from Sigma-Aldrich and all of the media were obtained from Duchefa (Harlem, The Netherlands).

### 4.2. Amino Acid Sequence Alignment and Phylogenetic Analysis of OsCADs

The amino acid sequences of OsCADs and functional CADs from other plant species were retrieved from the MSU RGAP database (http://rice.plantbiology.msu.edu/) [38] and the National Center for Biotechnological Information (https://www.ncbi.nlm.nih.gov/) database, respectively. Multiple amino acid sequence alignment was performed with Clustal-W [72], and a phylogenetic analysis was conducted with MEGA ver. 6 [73] using the neighbor-joining method. The amino acid sequences (accession numbers in parentheses) used in the alignment and phylogenetic analysis were AtCAD1 (AY288079), AtCAD2 (AY302077), AtCAD3 (AY302078), AtCAD4 (AY302081), AtCAD5 (AY302082), AtCAD6 (AY302075), AtCAD7 (AY302079), AtCAD8 (AY302080) and AtCAD9 (AY302076) from *A. thaliana*; EgCAD2 (CAA46585) from *E. gunnii*; NtCAD1 (CAA44216) and NtCAD2 (CAA44217) from *N. tabacum*; PtaCAD1 (CAA86073) from *P. taeda*; SbCAD2 (AEM63608) and SbCAD4 (XP_002462348) from *S. bicolor*; ZmCAD1 (CAA06687) and ZmCAD2 (CAA74070) from *Z. mays*; BdCAD3 (AFK80371) and BdCAD5 (AFK80372) from *B. distachyon*; TaCAD12 (ANW09910) from *T. aestivum*; PtrSAD (1YQD) from *P. tremuloides*; and PtoCAD12 (KJ159967) from *P. tomentosa*.

### 4.3. Construction of OsCAD Expression Vectors

Rice leaves and stems in the booting and heading stages were collected to isolate the total RNA with RNAiso (Takara, Shiga, Japan). The first cDNA was synthesized from the total RNA using SuPrimeScript RT premix with an oligo dT primer (GeNet Bio, Daejeon, Korea). Cloning primers were designed according to the sequences obtained from the MSU RGAP database. The amplification primer sequences and polymerase chain reaction (PCR) conditions are given in Supplementary Table S2. OsCADs were amplified with Solg^TM^ Pfu DNA Polymerase (SolGent, Daejeon, Korea). The PCR products were eluted with a FavorPrepTM Gel/PCR purification kit (Favorgen, Ping-Tung, Taiwan) and subcloned into the pJET 1.2 blunt cloning vector (Thermo Scientific, Carlsbad, CA, USA). After sequence confirmation, each *OsCAD* was cut out with the appropriate restriction enzymes (Enzynomics, Daejeon, Korea) and inserted into the pET28a(+) vector (Novagen, Madison, WI, USA) to produce His-tagged recombinant proteins. The *OsCAD*/pET28a(+) constructs were transformed into *E. coli* BL21(DE3) cells for heterologous expression of *OsCAD*s.

### 4.4. Heterologous Expression and Purification of Recombinant OsCADs

The E. coli transformants were grown in LB medium containing kanamycin (25 µg/mL) at 37 °C until the OD_600_ was 0.6. For the induction of OsCAD protein expression, 0.1 mM isopropyl β-d-thiogalactopyranoside was added to the culture, and the culture was incubated at 18 °C for 16 h. After induction, the cells were harvested by centrifugation (5000× g for 15 min), and the cell pellets were resuspended in phosphate buffered saline (PBS, 137 mM NaCl, 2.7 mM KCl, 10 mM Na_2_HPO_4_, 2 mM KH_2_PO_4_) supplemented with lysozyme (1 mg/mL) and phenylmethylsulfonyl fluoride (1 mM). After sonication of the resuspended cells, the crude protein extracts were obtained by centrifugation (15,900× g for 20 min, 4 °C). The crude protein extract was mixed with Ni-NTA agarose beads (Qiagen, Hilden, Germany) and incubated at 4 °C for 2 h with agitation. For purification of recombinant proteins, the mixtures were packed into a chromatography column and washed three times with five-column volumes of 20 mM imidazole in Tris buffer (50 mM Tris, pH 8.0, 300 mM NaCl). The recombinant proteins were eluted with 50 to 150 mM imidazole in the Tris buffer. The eluted proteins were analyzed with SDS-PAGE.

### 4.5. CAD Activity Assay

OsCAD activity was measured according to the method of Wryambik and Grisebach [74]. The reaction mixture consisted of 300 μM NADPH, 30 μM hydroxycinnamaldehyde, and 10 μg of purified recombinant OsCAD protein in 100 mM sodium/potassium phosphate buffer (pH 6.25) to a total volume of 500 μL at 30 °C. The reaction was initiated by the addition of recombinant OsCAD protein. Decreases in A_340_ with the disappearance of NADPH and hydroxycinnamaldehyde were monitored for 10 min using a Cary 300 Bio UV/Vis-spectrophotometer (Varian, Mulgrave, Victoria, Australia). The substrate concentrations to determine *K*_M_ and *V*_max_ were 5–50 μM. The *K*_M_ and *V*_max_ values of the OsCAD catalyzed reactions were calculated by extrapolation from Lineweaver-Burk plots. The results are represented as the mean ± standard deviation.

### 4.6. In Silico Transcriptomic and qRT-PCR Analyses of OsCADs

The public microarray data of OsCAD genes under various biotic and abiotic stresses were downloaded from the Genevestigator plant biology database (https://genevestigator.com/gv/doc/intro_plant.jsp) [75]. Microarray data from UV-C treated rice were obtained from the transcriptomic analysis conducted by Park et al. (2013) [49]. Genes that showed more than two-fold changes in expression level, with a *p*-value of less than 0.05, were selected as differentially expressed genes. Heatmap expression patterns were generated with the normalized data using the Multi Experiment Viewer program (http://www.tm4.org/mev.html).

Total RNA extraction from UV-treated rice leaves and cDNA synthesis were conducted by the methods described above. qRT-PCR was performed using Prime Q-Mastermix (GeNet Bio, Daejeon, Korea) on an AriaMx real-time PCR system (Agilent, Santa Clara, CA, USA). Transcript levels were normalized by the rice ubiquitin 5 (*UBQ5*) transcripts. The ΔΔ*C*t method was applied to calculate expression levels [76]. Primers for the qRT-PCR are listed in Table S3. The primer for *OsCAD8* was designed for the region conserved on *OsCAD8A*-*D*. To assess the expression of *OsCADs* in different tissues, qRT-PCR analysis was performed on triplicate biological samples.

## 5. Conclusions

To delineate the role of substantially expressed *OsCAD*s*, OsCAD1*, *2*, *6*, and *7*, were cloned from rice tissues and heterologously expressed in *E. coli* in a His-tagged form. An activity assay of the recombinant OsCAD proteins with hydroxycinnamaldehyde substrates showed that OsCAD2, 6, and 7 have CAD activity, but OsCAD1 has no detectable catalytic activity. The kinetic parameters demonstrated that OsCAD2 is the most active enzyme among the examined OsCADs. The catalytic efficiency of OsCAD2 was comparable with that of known *bona fide* CADs from other plant species. *OsCAD2* was found to be preferentially expressed in actively lignifying tissues such as the root, stem, and panicle. These findings and the close phylogenetic relationship with *bona fide* CADs from other plants suggest that *OsCAD2* is the major OsCAD involved in developmental lignification in rice. Biochemically functional *OsCAD6* and *7* are Class II CADs, and their expression was relatively low compared with *OsCAD2* and confined to certain tissues (leaf sheath, stem, and panicle). Our in silico and qRT-PCR analyses showed that *OsCAD2* and *6* expression was stimulated by *Xoo* infection and UV-treatment. This result suggests that *OsCAD2* plays a *bona fide* role in lignification and also functions in defense response. Low expression levels of *OsCAD6* during normal growth and its stress-stimulated expression suggests that it is an inducible OsCAD and that participates in the defense response of rice to biotic and abiotic stresses.

## Figures and Tables

**Table 1:** Rice *CAD* and *CAD-like* gene family retrieved from the MSU RGAP database.

Locus ID	Name ^a^	Gene Description	ORF ^b^	Protein Size (aa) ^c^	Theoretical MW (kDa) ^d^
LOC_Os10g11810	OsCAD1	dehydrogenase, putative, expressed	1065	354	38.5
LOC_Os02g09490	OsCAD2	dehydrogenase, putative, expressed	1092	363	38.6
LOC_Os10g29470	OsCAD3	dehydrogenase, putative, expressed	1101	366	38.5
LOC_Os11g40690	OsCAD4	dehydrogenase, putative, expressed	1032	343	37.5
LOC_Os08g16910	OsCAD5	dehydrogenase, putative, expressed	999	332	35.0
LOC_Os04g15920	OsCAD6	dehydrogenase, putative, expressed	1083	360	39.1
LOC_Os04g52280	OsCAD7	dehydrogenase, putative, expressed	1140	379	39.6
LOC_Os09g23530	OsCAD8A	dehydrogenase, putative, expressed	1080	359	36.8
LOC_Os09g23540	OsCAD8B	dehydrogenase, putative, expressed	1311	436	45.6
LOC_Os09g23550	OsCAD8C	dehydrogenase, putative, expressed	1320	439	45.9
LOC_Os09g23560	OsCAD8D	dehydrogenase, putative, expressed	1089	362	37.2
LOC_Os03g12270	OsCAD9	dehydrogenase, putative, expressed	1089	362	37.9

^a^ Names of *OsCAD*s as given by Tobias and Chow [34]; ^b^ ORF; Open reading frame; ^c^ aa; Amino acid; ^d^ MW; Molecular weight.

**Table 2:** Kinetic parameters of recombinant OsCAD2, 6 and 7 catalyzed reactions toward hydroxycinnamaldehydes ^a^.

Enzyme	Substrate	*K*_M_ (μM)	*V*_max_ (μmol min^−1^ mg^−1^)	*k*_cat_ (min^−1^)	*k*_cat_/*K*_M_ (μM^−1^ min^−1^)
OsCAD2	*p*-Coumaraldehyde	9.36 ± 2.50	17.79 ± 0.51	831.55	92.43
	Coniferaldehyde	4.37 ± 0.85	38.67 ± 10.08	1808.07	429.29
	Sinapaldehyde	8.19 ± 2.62	52.86 ± 15.52	2471.41	321.14
	Cinnamaldehyde	5.30 ± 3.42	11.62 ± 3.85	543.15	142.14
OsCAD6	*p*-Coumaraldehyde	10.88 ± 4.29	0.11 ±0.01	4.54	0.45
	Coniferaldehyde	16.18 ± 4.49	0.217 ± 0.04	9.22	0.58
	Sinapaldehyde	10.72 ± 0.47	0.103 ± 0.01	4.40	0.41
	Cinnamaldehyde	28.25 ± 11.36	0.439 ± 0.13	18.66	0.69
OsCAD7	*p*-Coumaraldehyde	2.66 ± 0.49	0.09 ± 0.00	4.15	1.58
	Coniferaldehyde	11.44 ± 2.16	0.38 ± 0.04	17.09	1.52
	Sinapaldehyde	6.42 ± 1.77	0.17 ± 0.04	7.65	1.27
	Cinnamaldehyde	14.11 ± 4.00	0.25 ± 0.03	11.29	0.82

^a^ The results are the mean ± standard deviation of three replicated experiment.

## Supplementary Materials

The following are available online, Figure S1: Alignment of amino acid sequences of OsCADs and other plant CADs, Figure S2: Phylogenetic analysis of OsCADs and other plant CADs, Figure S3: Quantitative real-time PCR analysis of *OsCAD* gene expression in rice tissues from different developmental stages, Figure S4: In silico transcriptomic analysis of *OsCAD* gene expression in response to biotic stress, Figure S5: In silico transcriptomic analysis of *OsCAD* expression in response to abiotic stress, Table S1: Sequence homologies between the deduced amino acid sequences of the OsCADs and *bona fide* CADs from other plants, Table S2: Primer sequences and PCR conditions for the cloning of *OsCAD*s, Table S3: Primer sequences and PCR conditions for quantitative real-time PCR analysis of *OsCAD*s.
