# Supplementary material for: Biochemical Characterization of the Rice Cinnamyl Alcohol Dehydrogenase Gene Family
Source: Molecules. 2018 Oct 16;23(10):2659. doi: 10.3390/molecules23102659 (PMC6222663; doi:10.3390/molecules23102659)
Supplement: Supplementary file 1 [file molecules-23-02659-s001.pdf]

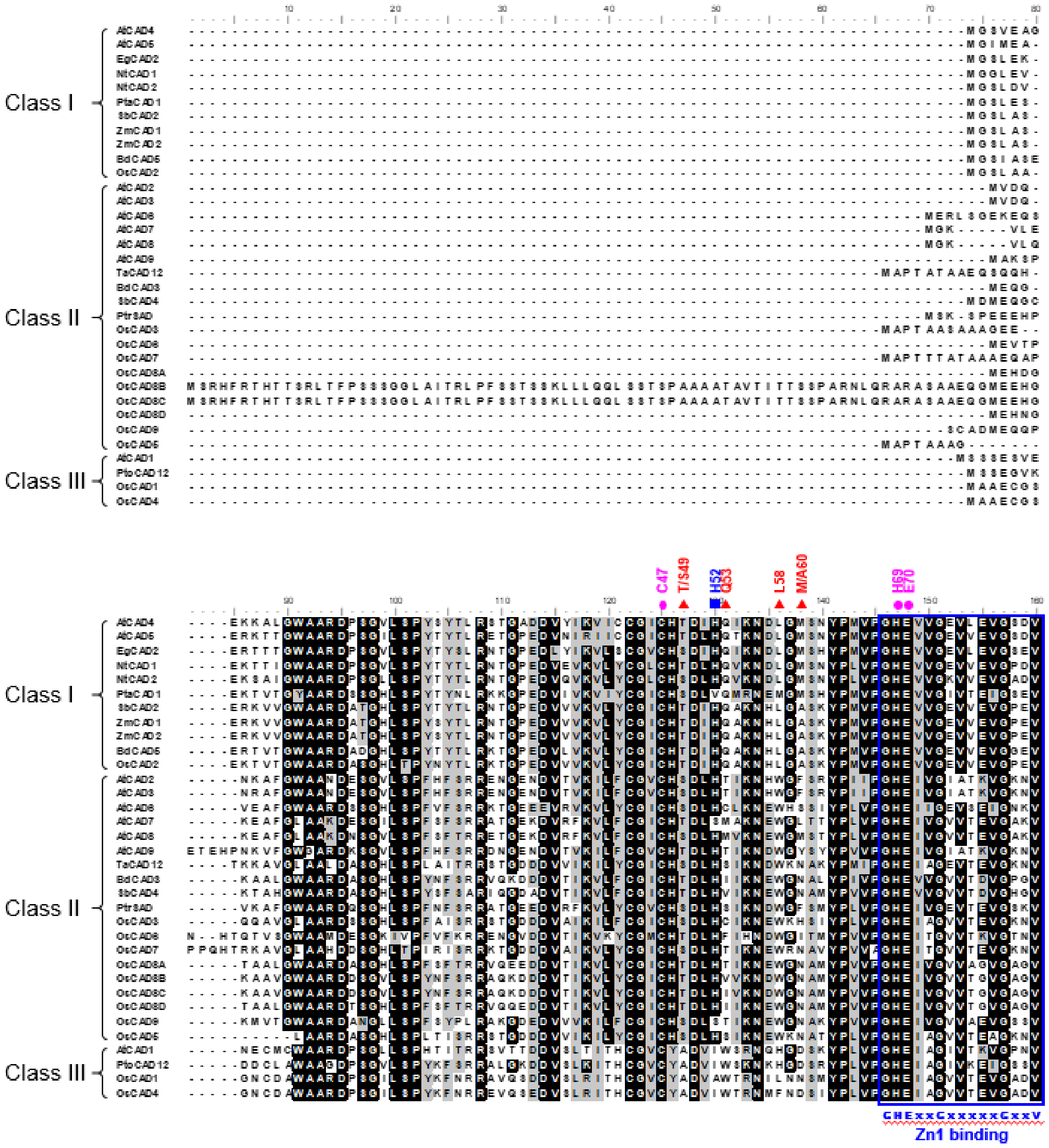
Class I
Class II
Class III
AtCAD4
AtCAD5
EgCAD2
NtCAD1
NtCAD2
PtaCAD1
SbCAD2
ZmCAD1
ZmCAD2
BdCAD5
OsCAD2
AtCAD2
AtCAD3
AtCAD6
AtCAD7
AtCAD8
AtCAD9
TaCAD12
BdCAD3
SbCAD4
PtrSAD
OsCAD3
OsCAD6
OsCAD7
OsCAD8A
OsCAD8B
OsCAD8C
OsCAD8D
OsCAD9
OsCAD5
AtCAD1
PtoCAD12
OsCAD1
OsCAD4
C47
T/S49
H52
Q53
L58
M/A60
H69
E70
CHExxCxxxxxCxxV
Zn1 binding

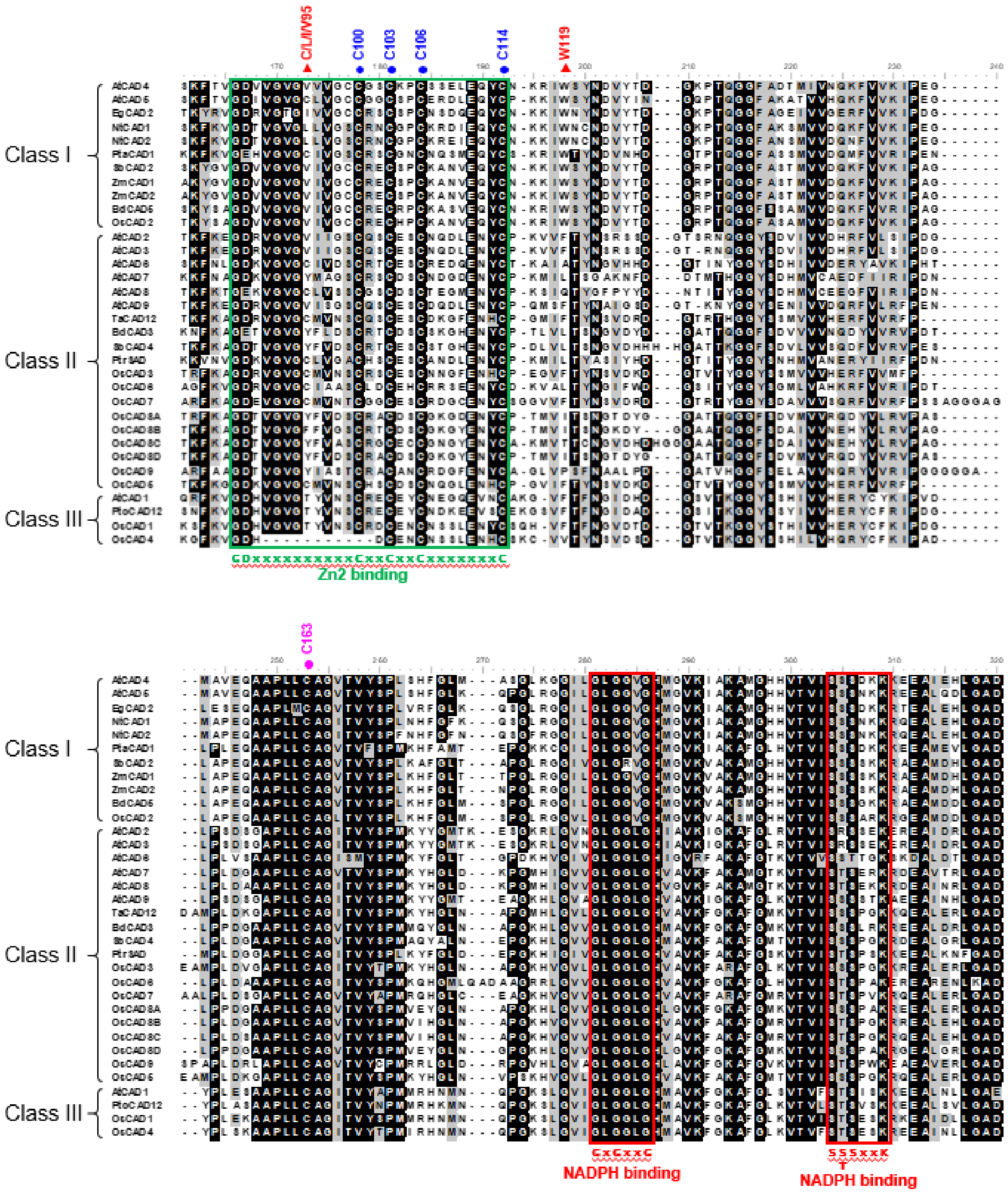
C/L/V95
C100
C103
C106
C114
W119
Class I
Class II
Class III
AtCAD4
AtCAD5
EgCAD2
NtCAD1
NtCAD2
PtaCAD1
SbCAD2
ZmCAD1
ZmCAD2
BdCAD5
OsCAD2
AtCAD2
AtCAD3
AtCAD6
AtCAD7
AtCAD8
AtCAD9
TaCAD12
BdCAD3
SbCAD4
PtrSAD
OsCAD3
OsCAD6
OsCAD7
OsCAD8A
OsCAD8B
OsCAD8C
OsCAD8D
OsCAD9
OsCAD5
AtCAD1
PtoCAD12
OsCAD1
OsCAD4
GDxxxxxxxxxxCxxCxxCxxxxxxxxC
Zn2 binding
C163
GxGxxG
NADPH binding
SSSxxK
NADPH binding

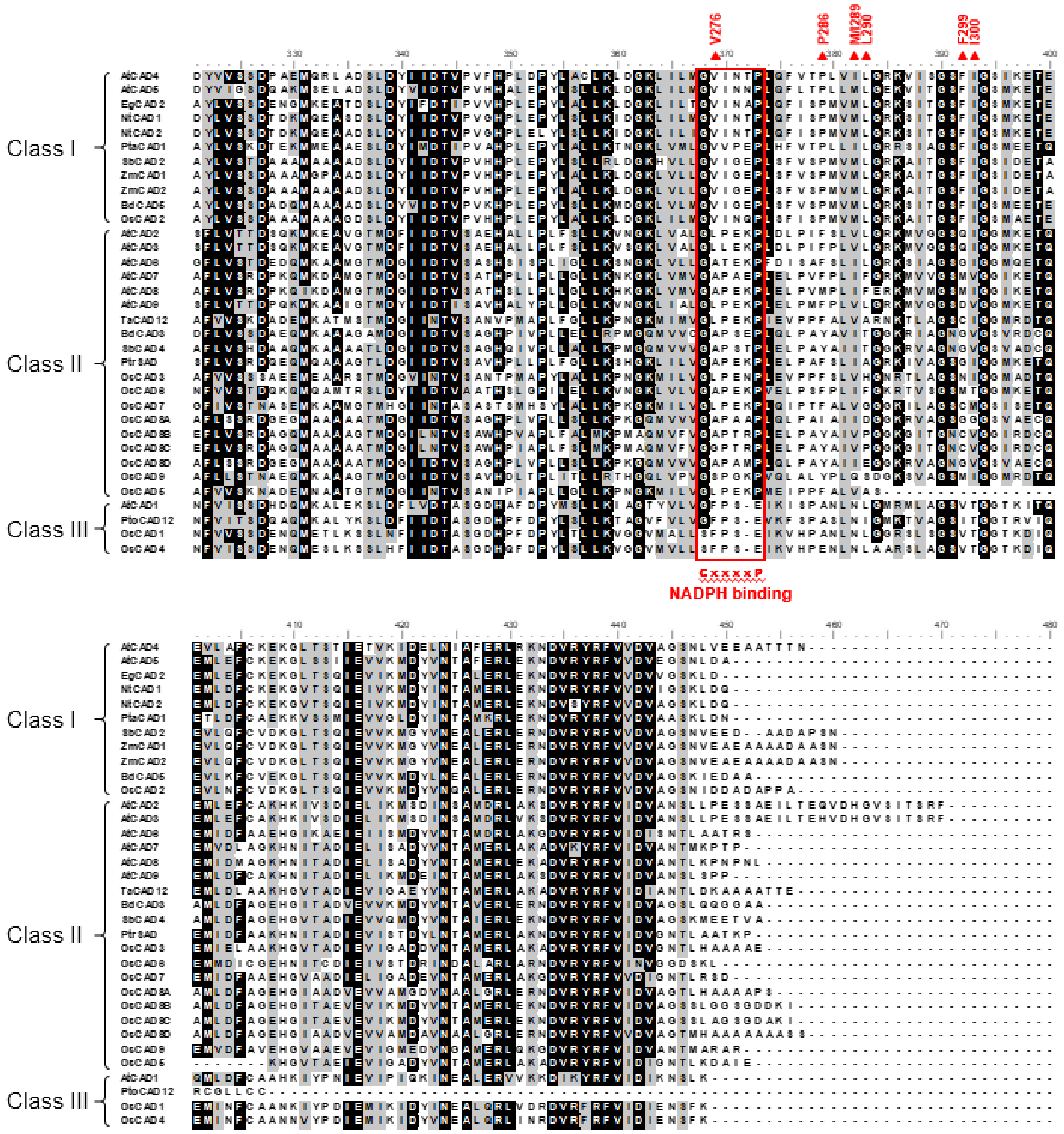

Figure S1. Alignment of amino acid sequences of OsCADs and other plant CADs. The shaded amino acids denoted identical or similar amino acids. The blue and green boxes indicate the Zn1 and Zn2 binding motives, respectively. The red boxes indicate NADPH binding motives. The consensus sequences are indicated below the aliments (x indicates any amino acid). Pink and blue circles indicate the conserved amino acids coordinated with the catalytic Zn^{2+} ion and the structural Zn^{2+} ion, respectively. Red triangles indicate the residues constituting the substrate binding pocket. Numbering of amino acids were follows AtCAD5. Amino acid sequences used are AtCAD1-9 from *A. thaliana*, EgCAD2 from *E. gunnii*, NtCAD1 and 2 from *N. tabacum*, PtaCAD1 from *P. taeda*, SbCAD2 and 4 from *S. bicolor*, ZmCAD1 and 2 from *Z. mays*, BdCAD3 and 5 from *B. distachyon*, TaCAD12 from *T. aestivum*, PtoCAD12 from *P. tomentosa*, and PtrSAD from *P. tremuloides*.

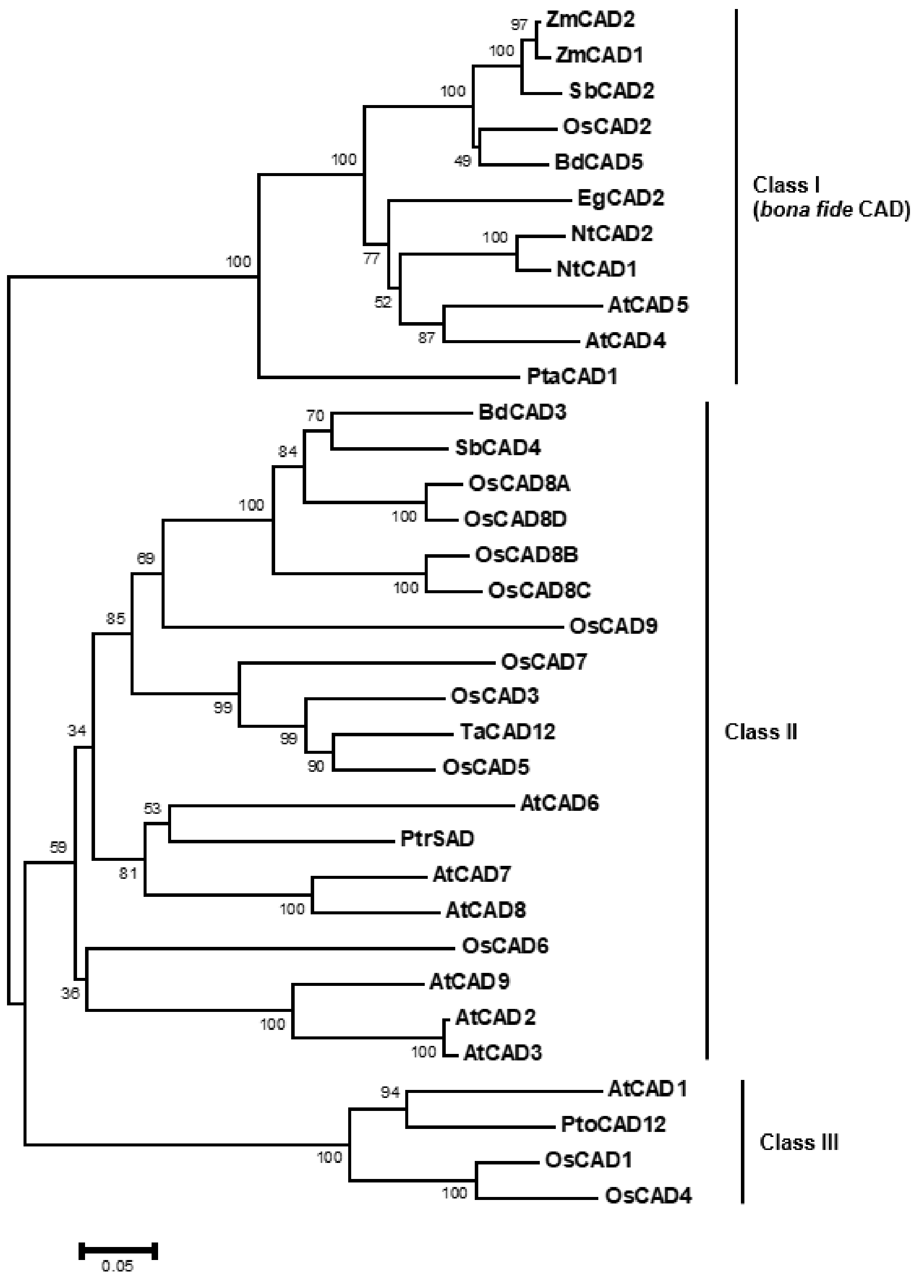

Figure S2. Phylogenetic analysis of OsCADs and other plant CADs. The phylogenetic tree was conducted using neighbor-joining criteria with the bootstrap values at 1,000 replicates using MEGA6. AtCAD1-9 from *A. thaliana*, EgCAD2 from *E. gunnii*, NtCAD1 and 2 from *N. tabacum*, PtaCAD1 from *P. taeda*, SbCAD2 and 4 from *S. bicolor*, ZmCAD1 and 2 from *Z. mays*, BdCAD3 and 5 from *B. distachyon*, TaCAD12 from *T. aestivum*, PtoCAD12 from *P. tomentosa*, and PtrSAD from *P. tremuloides*.

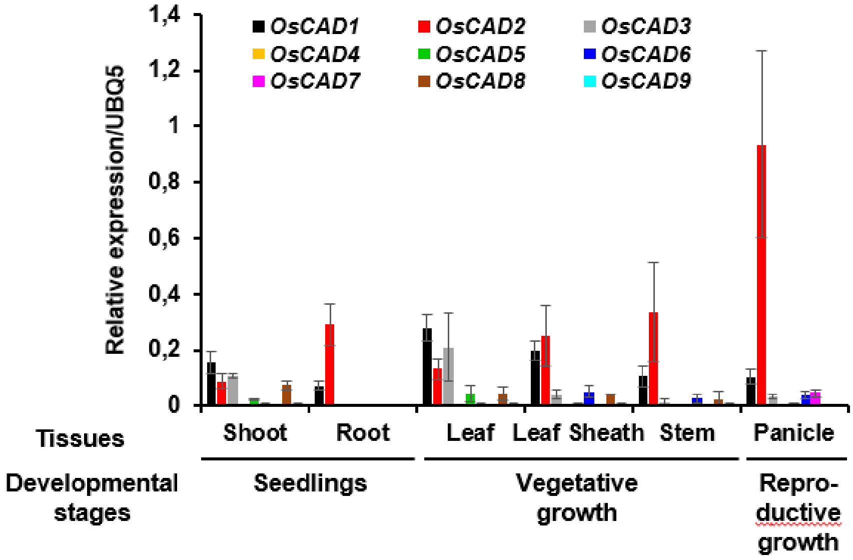

Figure S3. Quantitative real-time PCR analysis of *OsCAD* gene expression in rice tissues from different developmental stages. A ubiquitin gene (*OsUBQ5*) was amplified using specific primers and used as an internal control. Expression levels of each *OsCAD* gene are presented as the relative expression compared to *OsUBQ5*. The qRT-PCR analysis was performed on the triplicate biological samples.

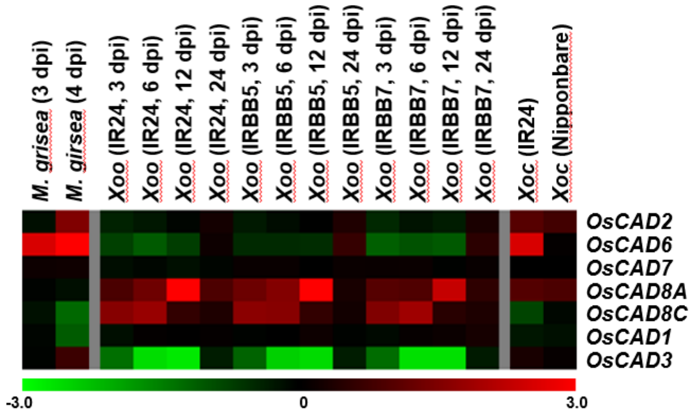

Figure S4. *In silico* transcriptomic analysis of *OsCAD* gene expression in response to biotic stress. The public microarray data of OsCAD genes were downloaded from the Genevestigator plant biology database (https://genevestigator.com/gv/doc/intro_plant.jsp). The expression patterns of *OsCADs* were analyzed by 3 and 4 days after infection of *Magnaporthe grisea*. The expression patterns of *OsCADs* in rice infected with different pathogenic lines of *Xanthomonas oryzae* pv. *oryzae* (*Xoo*) and *X. oryzae* pv. *oryzicola* (*Xoc*) are shown. The color scale represents the $\log_2$ fold changes of gene expression in response to *M. grisea, Xoo* and *Xoc* infections. Heatmaps were generated using the Multi Experiment Viewer program (http://www.tm4.org/mev.html).

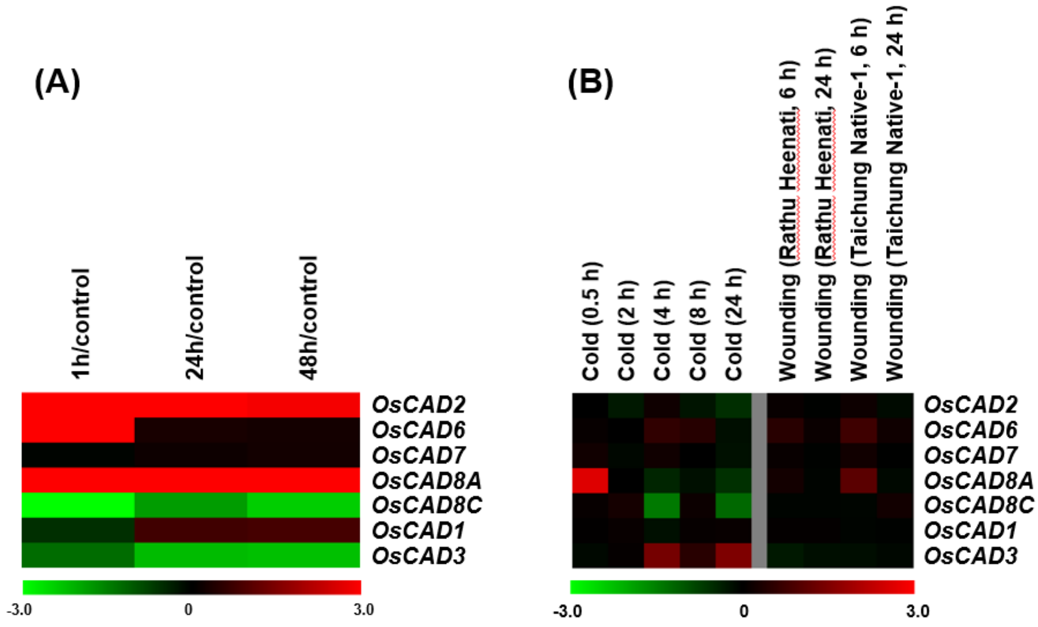

Figure S5. *In silico* transcriptomic analysis of *OsCAD* expression in response to abiotic stress. (A) The expression patterns of *OsCAD*s in UV-treated rice leaves. Microarray data previously obtained from rice leaves 1, 24 and 48 h after UV treatment (Park et al., 2013) were used for the analysis. (B) The expression patterns of *OsCCR*s in cold- and wounding-treated rice samples. Microarray data of cold- and wounding-treated rice samples were obtained from the Genevestigator plant biology database. The color scale represents the $\log_2$ fold changes of gene expression in response to abiotic stress conditions. Heatmaps were generated using the Multi Experiment Viewer program (http://www.tm4.org/mev.html).

Table S1. Sequence homologies between the deduced amino acid sequences of the OsCADs and *bona fide* CADs from other plants. The values show the percentage of amino acid identities and similarities. Left and right numbers indicate the identities and similarities, respectively.

	AtCAD4	AtCAD5	EgCAD2	NtCAD1	NtCAD2	PtaCAD1	SbCAD2	ZmCAD1	ZmCAD2
OsCAD1	44/64	45/64	46/65	46/65	46/65	47/68	42/61	42/61	42/61
OsCAD2	71/83	72/84	74/83	75/85	73/84	65/81	86/93	87/92	87/93
OsCAD3	45/64	46/63	47/64	46/65	46/64	49/67	43/62	44/63	44/63
OsCAD4	40/59	44/60	42/61	42/60	42/60	45/64	39/57	39/57	39/57
OsCAD5	42/57	44/60	44/59	45/60	45/60	46/65	42/58	42/59	42/59
OsCAD6	45/64	45/65	46/65	48/66	47/65	48/66	44/61	44/61	44/61
OsCAD7	43/62	43/62	44/60	43/62	44/61	45/65	41/59	41/59	42/59
OsCAD8A	48/63	47/63	47/63	49/65	50/66	49/67	50/63	50/63	51/64
OsCAD8B	40/56	39/55	42/54	42/57	42/58	40/57	41/54	40/54	41/54
OsCAD8C	40/56	40/55	41/54	43/57	43/58	40/56	41/54	40/54	41/54
OsCAD8D	48/63	47/62	47/62	49/64	50/65	49/67	50/63	51/64	51/64
OsCAD9	42/62	44/63	45/63	45/64	46/63	48/67	46/63	46/63	47/63

Table S2. Primer sequences and PCR conditions for the cloning of *OsCAD*s.

Gene	Primer sequence [a]	Annealing Temp. (°C)
OsCAD1	5′-CATATGGCTGCTGAATGTGGAAG-3′<br>5′-GTCGACCTACTTGAACGAGTTCTCAA-3′	50
OsCAD2	5′-CATATGGGCAGCCTCGCCGCCGA-3′<br>5′-GAATTCTCAGGCGGGCGGCGCGTCGG-3′	50
OsCAD6	5′-CATATGGAGGTCACCCCCAACCA-3′<br>5′-GAATTCCTAGAGCTTGGAGTCGCCCC-3′	50
OsCAD7	5′-CATATGGCGCCGACGACGACGGC-3′<br>5′-GAATTCTCAATCCGACCTGAGGGTGT-3′	50

Table S3. Primer sequences and PCR conditions for quantitative real-time PCR analysis of *OsCAD*s.

Gene	Primer sequence	Annealing Temp. (˚C)
OsCAD1	5′- GGCGGAGTTTATCTGGTAGT -3′<br>5′- AAATCTGACATCCGGTCAA -3′	55
OsCAD2	5′- TGTGTGAGACTCTGACGACTTGTC -3′<br>5′- CATATATTGCGAGGCCGAATTT -3′	55
OsCAD3	5′- CTCTGGCAGGGAGCAACATC -3′<br>5′- AAGCGATACCTGACATCCGC -3′	60
OsCAD4	5′- GCGACCATGACTGCGAGAAC -3′<br>5′- CCTTTGTTACGGTCCCGTCT -3′	60
OsCAD5	5′- CAGCATCAAGAACGAGTGGA -3′<br>5′- GAGTGGCACGAGTTCACCAT -3′	55
OsCAD6	5′- ATGACGAGGAGCCTTGACTA -3′<br>5′- CTCTTACCGAAGATGAGAGGG -3′	55
OsCAD7	5′- CATGCATGGCATCATCAACA -3′<br>5′- TAGTATCTTGCCCCCTCCAA -3′	60
OsCAD8	5′- TGGGGCAACGCCATGTACCC -3′<br>5′- AGCCCACGCCCACCGTGTCG -3′	60
OsCAD9	5′- ATCGGCGGGATGAGGGATA -3′<br>5′- GTACCTCACGTCTCCCTTCTG -3′	60
UBQ5	5′-ACCACTTCGACCGCCACTACT-3′<br>5′-ACGCCTAAGCCTGCTGGTT-3′	55/60
